# Cardiac Arrest Treatment Center Differences in Sedation and Analgesia Dosing During Targeted Temperature Management

**DOI:** 10.1007/s12028-022-01564-6

**Published:** 2022-07-28

**Authors:** Ameldina Ceric, Teresa L. May, Anna Lybeck, Tobias Cronberg, David B. Seder, Richard R. Riker, Christian Hassager, Jesper Kjaergaard, Zana Haxhija, Hans Friberg, Josef Dankiewicz, Niklas Nielsen

**Affiliations:** 1 Division of Anesthesia and Intensive Care, Department of Clinical Sciences Lund, Helsingborg Hospital, Lund University, Svartbrödragränden 3, 251 87 Helsingborg, Sweden; 2 Department of Critical Care, Maine Medical Center, Portland, ME USA; 3 Division of Anesthesia and Intensive Care, Department of Clinical Sciences Lund, Skane University Hospital, Lund University, Lund, Sweden; 4 Division of Neurology, Department of Clinical Sciences, Skane University Hospital, Lund University, Lund, Sweden; 5 Department of Cardiology, Rigshospitalet, University of Copenhagen, Copenhagen, Denmark; 6 Division of Anesthesia and Intensive Care, Department of Clinical Sciences Lund, Skane University Hospital, Lund University, Malmö, Sweden; 7 Division of Cardiology, Department of Clinical Sciences Lund, Skane University Hospital, Lund University, Lund, Sweden

**Keywords:** Cardiac arrest, Target temperature management, Analgosedation, Sedation, Analgesia, Seizures

## Abstract

**Background:**

Sedation and analgesia are recommended during targeted temperature management (TTM) after cardiac arrest, but there are few data to provide guidance on dosing to bedside clinicians. We evaluated differences in patient-level sedation and analgesia dosing in an international multicenter TTM trial to better characterize current practice and clinically important outcomes.

**Methods:**

A total 950 patients in the international TTM trial were randomly assigned to a TTM of 33 °C or 36 °C after resuscitation from cardiac arrest in 36 intensive care units. We recorded cumulative doses of sedative and analgesic drugs at 12, 24, and 48 h and normalized to midazolam and fentanyl equivalents. We compared number of medications used, dosing, and titration among centers by using multivariable models, including common severity of illness factors. We also compared dosing with time to awakening, incidence of clinical seizures, and survival.

**Results:**

A total of 614 patients at 18 centers were analyzed. Propofol (70%) and fentanyl (51%) were most frequently used. The average dosages of midazolam and fentanyl equivalents were 0.13 (0.07, 0.22) mg/kg/h and 1.16 (0.49, 1.81) µg/kg/h, respectively. There were significant differences in number of medications (*p* < 0.001), average dosages (*p* < 0.001), and titration at all time points between centers (*p* < 0.001), and the outcomes of patients in these centers were associated with all parameters described in the multivariate analysis, except for a difference in the titration of sedatives between 12 and 24 h (*p* = 0.40). There were associations between higher dosing at 48 h (*p* = 0.003, odds ratio [OR] 1.75) and increased titration of analgesics between 24 and 48 h (*p* = 0.005, OR 4.89) with awakening after 5 days, increased titration of sedatives between 24 and 48 h with awakening after 5 days (*p* < 0.001, OR > 100), and increased titration of sedatives between 24 and 48 h with a higher incidence of clinical seizures in the multivariate analysis (*p* = 0.04, OR 240). There were also significant associations between decreased titration of analgesics and survival at 6 months in the multivariate analysis (*p* = 0.048).

**Conclusions:**

There is significant variation in choice of drug, dosing, and titration when providing sedation and analgesics between centers. Sedation and analgesia dosing and titration were associated with delayed awakening, incidence of clinical seizures, and survival, but the causal relation of these findings cannot be proven.

**Supplementary Information:**

The online version contains supplementary material available at 10.1007/s12028-022-01564-6.

## Introduction

Cardiac arrest survivors undergo targeted temperature management (TTM) after resuscitation to reduce brain injury and improve the likelihood of a good functional outcome. This period of critical care involves dozens of clinical decisions including ventilatory strategy, hemodynamic targets, vasopressor support, organ support, and the provision of sedation and analgesia [1, 2]. Sedation and analgesia, which are almost universally provided to patients on life support and are required when patients are receiving neuromuscular blockade (NMB), have effects on patient comfort but also on hemodynamics, blood flow to the brain, and duration of mechanical ventilation, and they may reduce the occurrence of seizures and shivering during TTM [3–6]. Furthermore, it is also known that TTM alters pharmacodynamics and pharmacokinetics of most drugs including sedatives and analgesics [7–12]. Accumulation of these drugs delays awakening and confounds neurological prognostication [13]. Despite these important clinical effects, the optimal approach to analgosedation after cardiac arrest is not known.

Published guidelines give explicit recommendations on providing analgesia and sedation in the general medical and surgical intensive care unit (ICU) [14, 15], which includes frequent assessment of the level of arousal and the use of validated sedation scales. However, the postcardiac arrest pathophysiology makes applying those recommendations to this population problematic due to the effects of global brain injury, use of TTM, unstable hemodynamics, and interference with commonly used neuoprognositcation tools [16–20].

Determining best practices in sedation and analgesia after cardiac arrest begins with the knowledge of current standards of care. General protocols and practices to provide sedation and prevent shivering have been reported [21–26], but the specific medications, doses, and titration the individual patients receive in routine clinical practice are unknown. The main purpose of this study was to evaluate the average sedation and analgesia dosing adminstered to the individual patient cumulatively at 0–12, 12–24, and 24–48 h and titration between time points at 12–24 and 24–48 h between and within centers in a large international multicenter trial of temperature targets after cardiac arrest to characterize current practices. Secondly, we aimed to investigate clinically important outcomes that might be associated with that specific analgosedation pratices. We hypothesized there would be a significant variation in the specific analgosedation practices between and within centers and that this might be associated with the time to awakening, incidence of clinical seizures, or long-term survival.

## Material and Methods

### Patients

The TTM trial was an international, randomized, parallel group, assessor-blinded trial designed to evaluate outcome after TTM at either 33 °C (TTM33) or 36 °C (TTM36). The inclusion criteria of the TTM trial were patients 18 years of age or older who were unconscious (a score of < 8 on the Glasgow Coma Scale) on admission to the hospital after out-of-hospital cardiac arrest of presumed cardiac cause [27]. A center was defined as a study site in the TTM trial, i.e., an ICU, and it needed to be a high-volume center with percutaneous coronary intervention availability and the ability to provide TTM. Nine hundred fifty adult patients were enrolled from November 2010 to January 2013 within 4 h of return of spontaneous circulation (ROSC) at 36 ICUs in Europe and Australia. The 36 h of intervention consisted of achievement of target temperature, maintenance of target temperature, and rewarming to 37 °C. All patients were deeply sedated, endotracheally intubated, and mechanically ventilated. Survival at 6 months and good Cerebral Performance Category (CPC), defined as CPC 1 and 2, at 6 months were used in the analysis of this study. This study had ethical approval by the Regional Ethical Review Board Lund, Protocol 2009/6 Dnr 2009/324 (TTM Trial).

### Sedation and Analgesia

Approaches to sedation and analgesia were not defined in the study protocol. Centers were instructed to follow standard local practices and provide similar treatment to both intervention groups. The protocol specified that sedation should be stopped after 36 h of therapy to allow for assessment of awakening, unless required for medical reasons. Cumulative doses of sedative and analgesic drugs administered to each individual patient were collected by the treating center after primary data collection was complete [28]. This included doses of propofol, fentanyl, midazolam, morphine, remifentanil, alfentanil, sufentanil, and dexmedetomidine administered between 0 and 12 h, 12 and 24 h, and 24 and 48 h and were reported as cumulative doses of each drug type at 12, 24, and 48 h. The sedation depth may be monitored using clinical sedation assessment. The Richmond Agitation and Sedation Scale and the Critical Care Pain Observation Tool are two well-established, validated, and reliable sedation scales [29–31]. The Richmond Agitation and Sedation Scale functions by observing the patient and testing responsiveness to auditory and physical stimuli and the scale ranges from − 5 (unarousable), to 0 (alert and calm), to + 4 (combative) [29, 30]. The Critical Care Pain Observation Tool evaluates facial expression, muscle tension, movement, and compliance with ventilated breath/vocalized pain, with a total score ranging from 0 to 8 [31].

### Midazolam and Fentanyl Equivalents

Sedation and analgesia were separately normalized to midazolam and fentanyl equivalents. These conversions were based on the best available clinical and laboratory studies (see Supplement Table 1) [32–38]. Propofol was changed to midazolam equivalents and averaged over each time course and weight (in kilograms). Morphine, remifentanil, sufentanil, and alfentanil were converted to fentanyl equivalents and averaged over each time course and weight (in kilograms). Dexmedetomidine was not included, as it was only used by one site for one patient, and because there is no standard approach to conversion to midazolam equivalents.

### Awakening and Clinical Seizures

Awakening was defined as the first time the patient achieved a Glasgow Coma Scale motor subscore of 6. Level of consciousness was evaluated daily by using the Glasgow Coma Scale at all sites. Inclusion day was registered as day one, and late awakening was defined as a patient being awake after day five. Neuroprognostication according to study protocol was scheduled at 72 h after rewarming (108 h after ROSC) in patients who remained unconscious with strict criteria for withdrawal of life-sustaining therapy (WLST) [39]. We analyzed the number of patients who were awake within the first 48 h. Clinical seizure is defined as myoclonic or tonic–clonic seizures at any time point during the ICU stay.

### Missing Data

Only patients with sedation and analgesia data available were used in the primary analyses. To ensure the center practices are represented in the analysis for the aim of this study, we excluded centers with data for less than ten patients. We described the difference between patients with and without recorded sedation and analgesia data available by using the Wilcoxon rank-sum test, *χ*^2^ test, and Fisher’s exact test.

### Analysis

Cumulative dose of each medication was calculated for all patients and summarized across all centers at each time point. Propofol was converted to midazolam equivalents in mg/kg/h and analgesics were converted to fentanyl equivalents in µg/kg/h, to be able to compare the dosing of all patients in the cohort. Continuous data are expressed as medians and interquartile ranges unless otherwise indicated.

We evaluated the number of sedative and analgesic medications using analysis of variance (ANOVA) to test for global differences between centers. To analyze the association between the use of two or more sedatives and treatment center with adjustment for baseline severity of illness, we created a multivariate model using the clinically important and design variables of the TTM trial (age, sex, witnessed arrest, shockable rhythm, time to ROSC, and shock on admission).

To adjust for baseline severity of illness, clinical factors potentially affecting the delivered dose equivalents of sedatives and analgesics were tested with a linear regression model including clinically important design variables and the target temperature of 36 °C at 12, 24, and 48 h. The center was then added to this model and the two models were compared using *R*^2^ values and likelihood ratio testing.

Titration of sedation and analgesia was evaluated by using differences in patients’ average hourly dosages between 12–24 h and 24–48 h. This was again evaluated with adjustment for baseline severity of illness using the clinically important, design variables, and the target temperature of 36 °C. The center was then added to this model and the two models were compared using likelihood ratio testing. Survival at 6 months was then added to the multivariate model to test the association of sedative and analgesia titration.

The association of sedation and analgesia dosing at 12, 24, and 48 h, on a “center” level, with late awakening and clinical seizures were evaluated using a hierarchical logistic regression model. To adjust for baseline severity of illness, clinically important and design variables of the TTM trial were added to the analyses.

The association of sedation and analgesia titration between 12–24 h and 24–48 h, on a “center” level, with late awakening and clinical seizures were evaluated using a hierarchical logistic regression model. To adjust for baseline severity of illness, clinically important, design variables of the TTM trial, and target temperature of 36 °C were added to the analyses.

## Results

Among 36 centers, 21 participated in collecting sedation and analgesia data. Three centers enrolled less than ten patients and were excluded; therefore, nine patients were excluded. This left 18 centers with 614 patients to include in this study (see flowchart in Supplement Fig. 1). The proportion of patients enrolled in this study at each center ranged from 2 to 7% for 17 centers, and one center enrolled 26% of all patients. Fifteen centers out of 18 were university hospitals, and the other 3 centers were regional hospitals. One center and 163 (26%) patients were treated at a cardiac ICU and the others were treated at mixed ICUs. Most of the centers and patients (15 centers and 533 [87%] patients) used the Richmond Agitation-Sedation Scale. One center including 29 (4%) patients used Critical Care Pain Observation Tool. One center including 27 (4%) patients reported that no scale was used. One center including 29 (4%) patients did not report whether a sedation scale was used. Patient characteristics and the frequency of sedation and analgesic used are shown in Tables 1 and 2, respectively.

**Table 1:** Patient characteristics

Patient characteristics	*N* = 614^a^
Age	65 (56, 72)
Female sex	113 (18%)
Arrest at home	328 (53%)
Bystander witnessed	546 (89%)
Bystander CPR	444 (72%)
Bystander defibrillation	55 (9.0%)
Shockable rhythm	498 (81%)
Number of defibrillations	2 (1, 4)
Prehospital intubation	426 (70%)
Time to ROSC (min)	25 (16, 39)
Good CPC at 6 months	303 (49%)

^a^Statistics presented: median (IQR); *n* (%)

**Table 2:** Type of sedative and analgesic drugs given within the first 12, 24, and 48 h

Medication	Proportion: 12 h *n* (%)	Dose: median (IQR)	Proportion: 24 h *n* (%)	Dose: median (IQR)	Proportion: 48 h *n* (%)	Dose: median (IQR)
Propofol (mg/kg/h)	421 (69)	2.3 (1.2, 3.8)	431 (70)	2.4 (1.4, 4.3)	432 (70)	2.2 (1.1, 3.7)
Midazolam (mg/kg/h)	244 (40)	0.07 (0.04, 0.13)	259 (42)	0.09 (0.05, 0.10)	258 (42)	0.06 (0.03, 0.10)
Fentanyl (mcg/kg/h)	304 (50)	1.7 (1.2, 2.3)	310 (50)	1.9 (1.3, 2.4)	311 (51)	1.6 (1.1, 2.1)
Morphine (mg/kg/h)	96 (16)	0.04 (0.02, 0.05)	101 (16)	0.04 (0.02, 0.05)	101 (16)	0.3 (0.01, 0.04)
Remifentanil (mcg/kg/h)	84 (14)	3.3 (2.0, 6.1)	87 (14)	3.5 (2.3, 6.4)	84 (14)	3.7 (2.7, 6.0)
Alfentanil (mcg/kg/min)	32 (5)	29.3 (22.5, 37.4)	31(5)	33.3 (28.3, 37.0)	15 (2)	27.8 (23.7, 33.2)
Sufentanil (mcg/kg/h)	16 (3)	0.2 (0.1, 0.3)	16 (3)	0.2 (0.2, 0.3)	18 (3)	0.2 (0.1, 0.2)

A total of 325 patients from 18 centers in the TTM trial did not have sedation data or had less than ten patients at each center recorded and were not part of the cohort. These patients were less likely to have a good CPC at 180 days (Supplement Table 2).

### Number of Sedatives and Analgesia Medications Used

A total of 605 (99%), 607 (99%), and 607 (99%) patients had received a sedative at 12, 24, and 48 h, respectively, whereas 519 (85%), 527 (86%), and 508 (83%) patients had received an analgesic medication at 12, 24, and 48 h, respectively. No patient received more than two sedatives or analgesic medications. We found significant differences between centers in the number of sedatives and analgesics administered for all time points (*p* < 0.01 for all using grouped testing ANOVA). This remained consistent after the model was adjusted for age, sex, time to ROSC, bystander CPR, shockable rhythm, and shock on admission (*p* < 0.001 for grouped testing using ANOVA).

### Sedative and Analgesic Dosage

**Fig. 1:**
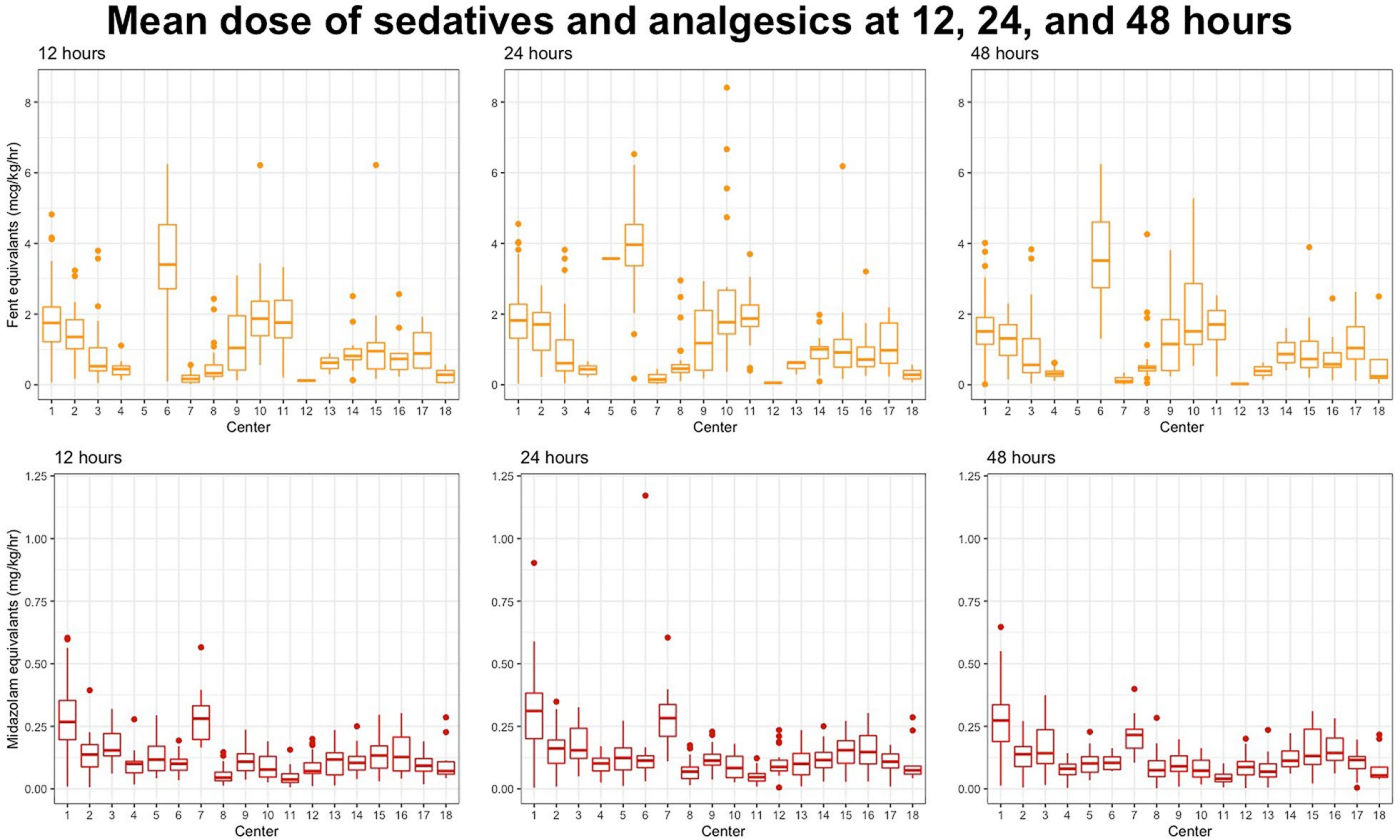
Illustrating the range of average doses of fentanyl (yellow) and midazolam (red) equivalents administered to patients in each center at 12, 24, and 48 h of treatment (colour figure online)

After normalizing the sedative dosages to midazolam equivalents, the median (interquartile range) dosages were 0.13 (0.08, 0.23), 0.14 (0.09, 0.24), and 0.13 (0.07, 0.22) mg/kg/h at 12, 24, and 48 h, respectively. The median (interquartile range) dosages of fentanyl equivalents were 1.21 (0.50, 2.04), 1.31 (0.52, 2.01), and 1.16 (0.49, 1.81) µg/kg/h at 12, 24, and 48 h, respectively. The dosages of fentanyl and midazolam equivalents, by center, at 12, 24, and 48 h are shown in Fig. 1. Linear models for associations with average sedative and analgesic dosages, adjusted for severity of illness and target temperature, with and without the center added to the model are shown in Table 3. Comparison of these models with likelihood ratio testing showed the center significantly improved model performance in both sedation and analgesia and at every time point (*p* < 0.001, for all models). The center effect significantly improved the *R*^2^ values for analgesics and sedation at all time points compared with models without the center effect (see Table 3). Target temperature was not significantly associated with average dosage of sedation or analgesia at any time point in multivariate model (see Table 3).

**Table 3:** Association of clinical factors, target temperature and center with average doses of fentanyl and midazolam equivalents in regression model with and without center, at 12, 24, and 48 h

Patient characteristics and *R*-square values	Sedation and analgesia	12 h	12 h with center	24 h	24 h with center	48 h	48 h with center
Age^a^	Fentanyl equivalents	− 0.05 (*p* = 0.009)	− 0.04 (*p* = 0.009)	− 0.07 (*p* = 0.003)	− 0.04 (*p* = 0.006)	− 0.04 (*p* = 0.04)	− 0.03 (*p* = 0.03)
	Midazolam equivalents	− 0.01 (*p* = 0.001)	− 0.01 (*p* < 0.001)	− 0.01 (*p* < 0.001)	− 0.01 (*p* < 0.001)	− 0.01 (*p* < 0.001)	− 0.01 (*p* < 0.001)
Female sex	Fentanyl equivalents	− 0.04 (*p* = 0.74)	− 0.12 (*p* = 0.22)	− 0.03 (*p* = 0.86)	− 0.08 (*p* = 0.44)	0.02 (*p* = 0.84)	− 0.08 (*p* = 0.39)
	Midazolam equivalents	− 0.1 (*p* < 0.01)	0.00 (*p* = 0.80)	− 0.02 (*p* = 0.14)	0.00 (*p* = 0.83)	− 0.01 (*p* = 0.25)	0.00 (*p* = 0.73)
Witnessed arrest	Fentanyl equivalents	− 0.06 (*p* = 0.71)	0.01 (*p* = 0.99)	− 0.01 (*p* = 0.97)	− 0.03 (*p* = 0.81)	0.06 (*p* = 0.73)	0.05 (*p* = 0.69)
	Midazolam equivalents	0.00 (*p* = 0.87)	0.00 (*p* = 0.97)	0.01 (*p* = 0.51)	0.00 (*p* = 0.73)	0.01 (*p* = 0.28)	0.01 (*p* = 0.41)
Shockable rhythm	Fentanyl equivalents	0.39 (*p* = 0.003)	0.16 (*p* = 0.11)	0.49 (*p* < 0.001)	0.25 (*p* = 0.02)	0.44 (*p* < 0.001)	0.24 (*p* = 0.009)
	Midazolam equivalents	0.02 (*p* = 0.05)	0.01 (*p* = 0.34)	0.03 (*p* = 0.01)	0.02 (*p* = 0.08)	0.03 (*p* = 0.007)	0.02 (*p* = 0.02)
Time to ROSC^b^	Fentanyl equivalents	− 0.2 (*p* = 0.03)	− 0.01 (*p* = 0.27)	− 0.03 (*p* = 0.01)	− 0.01 (*p* = 0.21)	− 0.02 (*p* = 0.046)	− 0.01 (*p* = 0.34)
	Midazolam equivalents	0.00 (*p* < 0.001)	0.00 (*p* < 0.001)	0.00 (*p* < 0.001)	0.00 (*p* < 0.001)	0.00 (*p* < 0.001)	0.00 (*p* = 0.004)
Shock on admission	Fentanyl equivalents	0.70 (*p* < 0.001)	0.35 (*p* = 0.006)	0.57 (*p* = 0.002)	0.23 (*p* = 0.10)	0.58 (*p* < 0.001)	0.27 (*p* = 0.03)
	Midazolam equivalents	− 0.02 (*p* = 0.22)	− 0.02 (*p* = 0.08)	− 0.01 (*p* = 0.65)	− 0.01 (*p* = 0.46)	− 0.02 (*p* = 0.22)	− 0.03 (*p* = 0.02)
Target temperature 36 °C	Fentanyl equivalents	0.03 (*p* = 0.78)	− 0.08 (*p* = 0.26)	0.08 (*p* = 0.45)	0.00 (*p* > 0.99)	0.09 (*p* = 0.33)	− 0.05 (*p* = 0.46)
	Midazolam equivalents	− 0.01 (*p* = 0.31)	0.01 (*p* = 0.17)	− 0.01 (*p* = 0.59)	− 0.01 (*p* = 0.43)	0.00 (*p* = 0.88)	0.00 (*p* = 0.84)
Center effect^c^	Fentanyl equivalents		***p* < 0.001**		***p* < 0.001**		***p* < 0.001**
	Midazolam equivalents		***p* < 0.001**		***p* < 0.001**		***p* < 0.001**
R-square values for the model	Fentanyl equivalents	0.07	0.51	0.08	0.53	0.07	0.52
	Midazolam equivalents	0.09	0.55	0.08	0.49	0.10	0.55

significant p-value with the significance level of 0.05 are in bold.

^a^Age estimate is per 5 year intervals

^b^Time to ROSC estimate is per 5 min intervals

^c^Center effect of global *p* value using ANOVA testing

### Titration of Sedatives and Analgesics

The median difference in midazolam dosing between 12 and 24 h was 0.009 (− 0.002 to 0.027) mg/kg/h and − 0.015 (− 0.042 to 0.001) mg/kg/h between 24 and 48 h. For fentanyl equivalents, the difference was 0.06 (− 0.01 to 0.22) mg/kg/h and − 0.14 (− 0.40 to 0.00) mg/kg/h between 12–24 and 24–48 h, respectively. Linear models for associations with differences in sedative and analgesic dosages, adjusted for severity of illness and target temperature, with and without the center added to the model are shown in Table 4. Comparison of these models with likelihood ratio testing showed the center significantly improved the models for sedation differences between 24 and 48 h (*p* < 0.001) and for differences in fentanyl dosage between 12 and 24 h (*p* = 0.04) and 24–48 h (*p* < 0.001). The center did not significantly affect the model for differences in midazolam equivalents between 12 and 24 h (*p* = 0.40). When survival was added to the model, survival at 6 months was associated with decreasing dosage between 24 and 48 h for fentanyl equivalents (*p* = 0.048) but not for midazolam equivalents (*p* = 0.75). Decreased titration between 12 and 24 h of midazolam and fentanyl equivalents were not associated with survival at 6 months (*p* = 0.06 and *p* = 0.38, respectively). Target temperature was significantly associated with increased dosage of fentanyl equivalents between 12 and 24 h (*p* = 0.08 and *p* = 0.02, without and with center effect, respectively), but there was no significant different between 24 and 48 h (see Table 4). Target temperature was not significantly associated with titration of midazolam equivalents at any time point.

**Table 4:** Association of clinical factors, center, and target temperature with difference in fentanyl and midazolam equivalent doses between 12–24 and 24–48 h with and without center

Patient characteristics	Sedation and analgesia	12–24 h difference	12–24 h difference with center	24–48 h difference	24–48 h difference with center
Age^a^	Fentanyl equivalents	0.00 (*p* = 0.82)	0.00 (*p* = 0.72)	0.01 (*p* = 0.38)	0.00 (*p* = 0.56)
	Midazolam equivalents	0.00 (*p* = 0.20)	0.00 (*p* = 0.13)	− 3.01 (*p* < 0.01)	− 1.83 (*p* = 0.01)
Female sex	Fentanyl equivalents	0.07 (*p* = 0.10)	0.06 (*p* = 0.99)	− 0.01 (*p* = 0.88)	− 0.04 (*p* = 0.49)
	Midazolam equivalents	− 0.01 (*p* = 0.38)	0.00 (*p* = 0.49)	− 0.61 (*p* = 0.91)	2.09 (*p* = 0.65)
Witnessed arrest	Fentanyl equivalents	0.10 (*p* = 0.08)	0.10 (*p* = 0.09)	0.04 (*p* = 0.56)	0.05 (*p* = 0.47)
	Midazolam equivalents	0.01 (*p* = 0.30)	0.00 (*p* = 0.57)	0.03 (*p* > 0.99)	6.92 (*p* = 0.27)
Shockable rhythm	Fentanyl equivalents	0.13 (*p* = 0.005)	0.11 (*p* = 0.01)	− 0.07 (*p* = 0.21)	− 0.03 (*p* = 0.61)
	Midazolam equivalents	0.01 (*p* = 0.08)	0.01 (*p* = 0.13)	13.0 (*p* = 0.02)	8.23 (*p* = 0.08)
Time to ROSC^b^	Fentanyl equivalents	0.00 (*p* = 0.38)	0.00 (*p* = 0.28)	0.01 (*p* = 0.30)	0.00 (*p* = 0.66)
	Midazolam equivalents	0.00 (*p* = 0.48)	0.00 (*p* = 0.61)	− 0.30 (*p* < 0.01)	0.002 (*p* = 0.44)
Shock on admission	Fentanyl equivalents	− 0.02 (*p* = 0.73)	− 0.05 (*p* = 0.38)	− 0.07 (*p* = 0.33)	− 0.01 (*p* = 0.89)
	Midazolam equivalents	0.01 (*p* = 0.18)	0.01 (*p* = 0.26)	− 7.77 (*p* = 0.25)	− 11.4 (*p* = 0.049)
Target temperature at 36 °C	Fentanyl equivalents	**0.09 (*p* = 0.01)**	**0.08 (*p* = 0.02)**	− 0.03 (*p* = 0.52)	− 0.04 (*p* = 0.36)
	Midazolam equivalents	0.01 (*p* = 0.27)	0.00 (*p* = 0.36)	1.41 (*p* = 0.73)	2.97 (*p* = 0.39)
Center effect^c^	Fentanyl equivalents		***p* = 0.048**		***p* < 0.001**
	Midazolam equivalents		*p* = 0.44		***p* < 0.01**

significant p-value with the significance level of 0.05 are in bold.

^a^Age estimate is per 5 year intervals

^b^Time to ROSC estimate is per 5 min intervals

^c^Center effect of global *p* value using ANOVA testing

To evaluate the effect of centers by country, the centers were clustered as follows: Switzerland (two centers), Denmark (one center), Italy (three centers), Luxemburg (one center), Netherlands (two centers), Norway (one center), Sweden (three centers), and United Kingdom (five centers). We evaluated the dosing characteristics (type of medication, number of medications, average dosage) at 24 h as well as dosing differences at 12 and 24 h were largely nonsignificant, and those that were significant did not differ substantially from the cohort.

### Awakening and Clinical Seizures

A total of 364 patients were alive at the end of the study period, of whom 342 had a registered day of awakening. Four patients (0.7%) the first day and 20 patients (3.3%) the second day of therapy were awake during the initial 48 h of therapy. We found a significant association of a higher average dosage of fentanyl received at 48 h with late awakening in multivariate analysis (*p* = 0.002 and *p* = 0.003 with and without center effect, respectively), whereas average dosage at 12 and 24 h were not significantly associated (see Supplement Table 3). Increase in titration dosing of fentanyl equivalents between 24 and 48 h was significantly associated with late awakening in multivariate analysis (*p* = 0.04 and *p* = 0.005 without and with center effect, respectively), shown in Supplement Table 4. Total dose of midazolam equivalents was not significantly associated with late awakening at any time point. Increased titration of midazolam equivalents between 24 and 48 h was significantly associated with late awakening in multivariate analysis (*p* < 0.001 for both with and without center effect), whereas titration between 12 and 24 were not (see Supplement Table 4).

Total average dosage of midazolam equivalents at any time point was not significantly associated with clinical seizures (see Supplement Table 5). An increase in titration of midazolam equivalent dosing between 24 and 48 h was significantly associated with clinical seizures (*p* = 0.04), whereas titration between 12 and 24 h was not associated (see Supplement Table 6). This finding remained consistent after adjustment for clinically relevant variables, target temperature, and the center effect. Neither average dose nor titration of fentanyl equivalents during the initial 48 h of therapy were associated with clinical seizures.

## Discussion

In a large randomized clinical trial population of patients receiving TTM after cardiac arrest, we found significant differences in the approach to providing sedation and analgesia. The level of target temperature was not significantly associated with total dose or titration of sedation and analgesics during the initial 48 h of therapy, except for increased titration of analgesics between 12 and 24 h with a target level of 36 °C. The treatment center was independently and strongly associated with the number of medications given, dosing, and titration of sedatives and analgesics, during and immediately following temperature management. Treatment center remained independently associated for most of the described parameters after adjustment for target temperature and clinically relevant variables, including markers of severity of illness like initial heart rhythm and total ischemic time. This suggests that local protocols influence sedation and analgesic dosing more than patient factors. We also found the total dose and titration of sedatives and analgesics to be associated with late awakening, clinical seizures, and survival, demonstrating the association of sedation and analgesia practices and clinically important outcomes. This study highlights the variability of sedation and analgesia practices between centers, implicating a gap of knowledge in optimal dosing and titration regimens and how this may affect the time to awakening and the incidence of clinical seizures and survival. To better determine whether the association of sedation and analgesia dosing or drug titration with clinically important outcomes is a causal one, further research in a prospective manner is needed.

We found that higher dosing at 48 h and increased dosing of analgesics between 24 and 48 h were associated with late awakening. Opioid analgesics may blunt the response to painful stimuli in the Glasgow Coma Scale and impair the pupillary light reflex, potentially affecting neurological prognostication. However, we did not find an association between average dosage of sedatives and time to awakening, which may be because most patients received a short-acting sedative (propofol 70%). These findings are in concordance with current guidelines, which advise the use of short-acting drugs (propofol, sufentanil, remifentanil) to shorten time to awakening and facilitate neurological prognostication [1]. The use of short-acting agents compared with long-acting agents (midazolam and fentanyl) has been associated with shorter duration of mechanical ventilation and earlier awakening, although no conclusion can be stated about impact on survival or neurological outcome [40–42]. Longer time to awakening makes patients susceptible to a perception of poor neurological prognosis and a premature withdrawal of life-sustaining therapies that can affect the ultimate outcome [43]. Further investigations of the effects of sedation and analgesics on neurological prognostication and outcome are warranted.

The need to control shivering in patients receiving TTM is important, regardless of the target temperature, and either escalation of sedation dosing or the use of NMB with a basal sedation dose is typically used to achieve this. It is required that adequate sedation is provided to all patients receiving NMB, as it is paramount for patient comfort. The incidence of shivering, and therefore NMB need, is related to the severity of brain injury, and escalating sedation dosing may be a reflection of attempts to control shivering. There were a small number of patients in our cohort who received no sedation. It is unclear whether these patients had severe brain injury and therefore did not require NMB or if they inappropriately received NMB without sedation. Although this information was not available in our data, the incidence, severity, and response to shivering should be closely evaluated to understand the effects of sedation dosing and titration.

Sedatives are antiepileptic and frequently used as treatment for clinical seizures during TTM; we speculate this may explain the association found in this study between increased sedative dosing between 24 and 48 h and clinical seizures [44–48]. We also found decreased dosing of analgesics to be associated with improved survival. Sedation and analgesia were mandatory during TTM for 36 h, and thus the dosing collected at 48 h reflect the dosing in patients with prolonged sedation. Prolonged sedation and analgesia may relate to increased shivering, which is associated with good outcome after cardiac arrest, but also pose a risk of secondary brain injury if not properly treated [49]. However, prolonged sedation and analgesia may also be due to patients having frequent myoclonus, indicating more severe brain injury and the need for continued sedation and analgesia [45]. Another common reason for prolonged sedation is the need for continued mechanical ventilation. These are possible reasons for the association found between dosing and titration with late awakening, the prevalence of clinical seizures and survival. Although sedatives may affect cerebral oxygen consumption, cerebral blood flow, and can suppress seizures, it remains unclear if sedation provides additional neuroprotective effects during TTM [3, 4, 17]. We speculate that the optimal dosage of sedatives needed during the post cardiac arrest care might depend on the severity of brain injury. Thus, sedation dosing should be individualized to the patient’s severity of brain injury, presence of myoclonus and shivering, and the intensity of shivering. These methods could be refined as future studies provide more information regarding phenotyping or better accounting for heterogeneity in this population.

The “center effect” is highlighted most prominently in analyzing the dosage of analgesia and sedatives. Here, the model without centers performed relatively poorly, with clinical factors accounting for only 7–10% of the variability of dosing differences (reflected in the *R*-squared). When “center” was added, the model was able to account for roughly half of the variability. Clearly, the dosing for these patients is complicated and there is still much work to be done to determine how bedside sedation decisions are being made. However, evaluation of single-center influence of sedation dosing, without the use of other physiologic end points, is of little use. It should be acknowledged that participating centers were high-volume cardiac arrest centers treating patients in the setting of a randomized trial. It is unknown if there would be similar findings in a “real world” setting across lower-volume institutions and outside of clinical trials. This variability should be further studied in larger cohorts that are powered to risk adjust for patient severity of illness to determine whether there is an individual effect on outcome for sedation and analgesia dosing on individual patients. Understanding this effect would inform trials to determine a synchronized approach to individualizing sedation and analgesia dosing.

A limitation of this study is that the primary objective of the TTM trial was not to investigate sedation and analgesia management, and thus dosing data were collected retrospectively. As such, not all centers participated, and data were collected up to 48 h although the trial intervention ended at 36 h. There were 623 out of 939 patients from the main trial with complete sedation and analgesia data, which may have introduced responder bias. We found a significantly lower rate of good CPC outcome, 42% compared with 49%, in patients without sedation data. This finding is a possible site effect because not all centers from the main TTM trial participated in this study. This study reports data collected more than 10 years ago from the TTM trial, another potential limitation of this study. However, this study is the first of our knowledge reporting individual patient sedation and analgesia data across several centers and these medications are still used in current practices. Postcardiac arrest organ dysfunction, specifically liver and kidney, may impact clearance of sedative and analgesic drugs and we not adjusted for, representing a limitation of this study. Although this is the largest study to evaluate the effects of sedation on outcomes after cardiac arrest, several limitation warrant discussions. As discussed above, the use of NMB and presence of shivering were not collected for this study. Given that sedation is required for patients receiving NMB, variation in the use of NMB and presence of shivering may have impacted the dosing and titration of sedatives and the relation to outcome. However, this may not only vary between centers but also between patients within centers depending on the severity of brain injury. Thus, the reasons for dosing and titration decisions were not available and should be further studied in a prospective manner to better understand the nuance of these differences. We also cannot determine causation in a retrospective study, so the associations we noted between sedation and analgesia dosing and titration with delayed awakening and clinical seizures at various time points should be considered as hypothesis-generated rather than definitive. The occurrence of subclinical seizures that may impact the sedation and analgesia dosing were not captured in this study, which is a limitation.

## Conclusions

We identified significant differences between centers in the choice of sedative and analgesic drugs, specific drug dosing, and titration during and immediately following TTM after out-of-hospital cardiac arrest. Higher dosages and upward titration of analgesics and sedatives during the initial 48 h of therapy were associated with delayed awakening and a higher incidence of clinical seizures. We also found that a downward titration of analgesics was significantly associated with survival at 6 months. The present study cannot assess the causal relation of the associations reported, and thus the findings are to be interpreted with caution. Clinical prospective trials comparing different regimens of sedations are needed to further elucidate these findings.

## Supplementary Information

Below is the link to the electronic supplementary material.

Supplementary file1 (DOCX 23 kb)

Supplementary file2 (DOCX 13 kb)

Supplementary file3 (DOCX 14 kb)

Supplementary file4 (DOCX 15 kb)

Supplementary file5 (DOCX 14 kb)

Supplementary file6 (DOCX 17 kb)

Supplementary file7 (DOCX 14 kb)
