# Supplementary material for: Cardiac Arrest Treatment Center Differences in Sedation and Analgesia Dosing During Targeted Temperature Management
Source: Neurocrit Care. 2022 Jul 28;38(1):16–25. doi: 10.1007/s12028-022-01564-6 (PMC9935704; doi:10.1007/s12028-022-01564-6)
Supplement: Supplementary file 1 — Supplementary file1 (DOCX 23 kb) [file 12028_2022_1564_MOESM1_ESM.docx]

Supplement figure 1. Flowchart. Flowchart of patients included and excluded in this substudy of the target temperature management trial (TTM-trial).

15 centers did not participate in collecting sedation and analgesia data, thus 316 patients were excluded

939 patients from 36 centers were included in the TTM-trial

623 patients from 21 centers

9 patients from 3 centers excluded due to few patients in each center

614 patients from 18 centers included in this substudy
