# Supplementary material for: Cardiac Arrest Treatment Center Differences in Sedation and Analgesia Dosing During Targeted Temperature Management
Source: Neurocrit Care. 2022 Jul 28;38(1):16–25. doi: 10.1007/s12028-022-01564-6 (PMC9935704; doi:10.1007/s12028-022-01564-6)
Supplement: Supplementary file 2 — Supplementary file2 (DOCX 13 kb) [file 12028_2022_1564_MOESM2_ESM.docx]

**Supplement table 1:** Equations for conversation to midazolam and fentanyl equivalents.

| Drugs | Equation |
| --- | --- |
| Propofol to midazolam equivalents | midazolam dose/number of hours/15 |
| Morphine to fentanyl equivalents | morphine dose/100/number of hours |
| Remifentanyl to fentanyl equivalents | remifent dose/number of hours/7 |
| Sufentanil to fentanyl equivalents | sufentanil dose/number of hours/0.167 |
| Alfentanil to fentanyl equivalents | alfentanil dose*0.125/number of hours |
