# Supplementary material for: Cardiac Arrest Treatment Center Differences in Sedation and Analgesia Dosing During Targeted Temperature Management
Source: Neurocrit Care. 2022 Jul 28;38(1):16–25. doi: 10.1007/s12028-022-01564-6 (PMC9935704; doi:10.1007/s12028-022-01564-6)
Supplement: Supplementary file 3 — Supplementary file3 (DOCX 14 kb) [file 12028_2022_1564_MOESM3_ESM.docx]

**Supplement table 2:** Table of patients with missing sedation data.

| **Patient and cardiac arrest characteristics** | **Missing**  N = 325^a^ | **Non-missing**  N = 614^a^ | **p-value^a^** | |
| --- | --- | --- | --- | --- |
| Age | 65 (57, 73) | 65 (56, 72) | 0.4 | |
| Female Sex | 65 (20%) | 113 (18%) | 0.5 | |
| Arrest at home | 171 (53%) | 329 (53%) | 0.8 | |
| Bystander witnessed | 291 (90%) | 547 (89%) | 0.7 | |
| Bystander CPR | 238 (73%) | 445 (72%) | 0.7 | |
| Bystander defibrillation | 38 (12%) | 55 (8.9%) | 0.2 | |
| Shockable rhythm | 253 (78%) | 499 (81%) | 0.3 | |
| Time to ROSC (minutes) | 27 (18, 40) | 25 (16, 39) | 0.13 | |
| Good CPC at 6 months | 136 (42%) | 304 (49%) | **0.03** | |
| *^a^*Statistics presented: median (IQR); n (%). Statistical tests performed: Wilcoxon rank-sum test; chi-square test of independence; Fisher's exact test | | | |  |
