# Supplementary material for: Cardiac Arrest Treatment Center Differences in Sedation and Analgesia Dosing During Targeted Temperature Management
Source: Neurocrit Care. 2022 Jul 28;38(1):16–25. doi: 10.1007/s12028-022-01564-6 (PMC9935704; doi:10.1007/s12028-022-01564-6)
Supplement: Supplementary file 4 — Supplementary file4 (DOCX 15 kb) [file 12028_2022_1564_MOESM4_ESM.docx]

**Supplement table 3:** Association of clinical factors and total doses of midazolam and fentanyl equivalents at 12, 24 and 48 hours with awakening after 5 days, without and with center effect, in multivariate analysis.

| Patient characteristics and medication | 12 hours | 12 with center | 24 hours | 24 with center | 48 hours | 48 with center |
| --- | --- | --- | --- | --- | --- | --- |
| Age^a^ | 1.02 (0.91, 1.14) P=0.70 | 1.05 (0.93, 1.19) p=0.40 | 1.04 (0.93, 1.16) p=0.50 | 1.07 (0.95, 1.20) p=0.30 | 1.02 (0.91, 1.14) p=0.70 | 1.05 (0.93, 1.19) p=0.40 |
| Female sex | 0.57 (0.22, 1.31) p=0.20 | 0.64 (0.26, 1.61) p=0.30 | 0.56 (0.22, 1.27) p=0.20 | 0.62 (0.25, 1.54) p=0.30 | 0.65 (0.25, 1.50) p=0.30 | 0.73 (0.29, 1.86) p=0.50 |
| Witnessed arrest | 1.01 (0.40, 2.78) p>0.9 | 1.25 (0.44, 3.58) p=0.70 | 0.94 (0.37, 2.59) p=0.90 | 1.16 (0.41, 3.29) p=0.80 | 1.00 (0.39, 2.80) p>0.9 | 1.22 (0.42, 3.54) p=0.70 |
| Shockable rhythm | 0.60 (0.21, 1.78) p=0.30 | 0.48 (0.16, 1.50) p=0.20 | 0.57 (0.20, 1.72) p=0.30 | 0.46 (0.15, 1.44) p=0.20 | 0.46 (0.16, 1.43) p=0.20 | 0.38 (0.12, 1.21) p=0.10 |
| Time to ROSC^b^ | 1.01 (0.99, 1.03) p=0.20 | 1.01 (1.00, 1.03) p=0.13 | 1.01 (1.00, 1.03) p=0.20 | 1.01 (1.00, 1.03) p=0.10 | 1.02 (1.00, 1.03) p=0.09 | 1.02 (1.00, 1.04) p=0.048 |
| Shock on admission | 3.56 (1.23, 10.7) p=0.02 | 3.76 (1.24, 11.4) p=0.02 | 3.92 (1.39, 11.7) p=0.01 | 4.36 (1.46, 13.0) p<0.01 | 2.77 (0.85, 9.36) p=0.09 | 3.14 (0.91, 10.8) p=0.07 |
| Fentanyl equivalents | 1.16 (0.90, 1.50) p=0.20 | 1.25 (0.90, 1.73) p=0.20 | 1.16 (0.93, 1.45) p=0.20 | 1.18 (0.89, 1.57) p=0.20 | **1.63 (1.20, 2.24) p=0.002** | **1.75 (1.21, 2.54) p=0.003** |
| Midazolam equivalents | 0.23 (0.02, 2.32) p=0.20 | 0.08 (0.00, 2.44) p=0.15 | 0.50 (0.07, 3.26) p=0.50 | 0.44 (0.04, 4.77) p=0.50 | 0.93 (0.08, 10.6) p>0.9 | 2.60 (0.09, 78.6) p=0.60 |
| ^a^Age estimate is per 5 year intervals  ^b^ Time to ROSC estimate is per 5 minute intervals | | | | | | |
