# Supplementary material for: Cardiac Arrest Treatment Center Differences in Sedation and Analgesia Dosing During Targeted Temperature Management
Source: Neurocrit Care. 2022 Jul 28;38(1):16–25. doi: 10.1007/s12028-022-01564-6 (PMC9935704; doi:10.1007/s12028-022-01564-6)
Supplement: Supplementary file 6 — Supplementary file6 (DOCX 17 kb) [file 12028_2022_1564_MOESM6_ESM.docx]

**Supplement table 5:** Association of clinical factors and total doses of midazolam and fentanyl equivalents at 12, 24 and 48 hours with clinical seizures, without and with center effect, in multivariate analysis.

| Patient characteristics and medication | 12 hours | 12 with center | 24 hours | 24 with center | 48 hours | 48 with center |
| --- | --- | --- | --- | --- | --- | --- |
| Age^a^ | 1.06 (0.97, 1.16) p=0.20 | 1.06 (0.97, 1.16) p=0.20 | 1.06 (0.97, 1.16) p=0.20 | 1.06 (0.97, 1.16) p=0.20 | 1.09 (1.00, 1.19) p=0.07 | 1.09 (0.99, 1.18) p=0.07 |
| Female sex | 0.95 (0.54, 1.64) p=0.90 | 1.04 (0.60, 1.82) p=0.90 | 0.91 (0.52, 1.58) p=0.70 | 1.09 (0.62, 1.92) p=0.80 | 1.01 (0.57, 1.75) p>0.9 | 0.99 (0.57, 1.74) p>0.90 |
| Witnessed arrest | 0.75 (0.39, 1.49) p=0.40 | 0.75 (0.38, 1.47) p=0.40 | 0.73 (0.38, 1.46) p=0.40 | 0.73 (0.37, 1.45) p0.40 | 0.76 (0.39, 1.52) p=0.40 | 0.76 (0.39, 1.49) p=0.40 |
| Shockable rhythm | 0.26 (0.15, 0.43) p<0.001 | 0.26 (0.15, 0.43) p<0.001 | 0.25 (0.15, 0.43) p<0.001 | 0.26 (0.15, 0.44) p<0.001 | 0..25 (0.15, 0.42) p<0.001 | 0.25 (0.15, 0.42) p<0.001 |
| Time to ROSC^b^ | 1.02 (1.01, 1.03) p<0.001 | 1.02 (1.01, 1.03) p<0.001 | 1.02 (1.01, 1.03) p<0.001 | 1.02 (1.01, 1.03) p<0.001 | - 1. (1.01, 1.03)   p<0.001 | 1.02 (1.01, 1.03)  p<0.001 |
| Shock on admission | 0.55 (0.24, 1.19) p=0.14 | 0.54 (0.24, 1.20) p=0.13 | 0.61 (0.28, 1.26) p=0.20 | 0.59 (0.27, 1.26) p=0.20 | 0.68 (0.30, 1.45) p=0.30 | 0.68 (0.31, 1.48) p=0.30 |
| Fentanyl equivalents | 0.90 (0.72, 1.10) p=0.30 | 0.89 (0.71, 1.11) p=0.30 | 0.90 (0.74, 1.09) p=0.30 | 0.90 (0.73, 1.12) p=0.30 | 1.04 (0.82, 1.30) p=0.70 | 1.04 (0.83, 1.30) p=0.70 |
| Midazolam equivalents | 1.37 (0.22, 8.21) p=0.70 | 0.93 (0.08, 10.7) p>0.90 | 1.35 (0.25, 6.68) p=0.70 | 0.70 (0.08, 6.41) p=0.80 | 2.55 (0.35, 18.2) p=0.40 | 2.55 (0.36, 18.3) p=0.40 |
| ^a^Age estimate is per 5 year intervals  ^b^ Time to ROSC estimate is per 5 minute intervals | | | | | | |
