# Supplementary material for: Cardiac Arrest Treatment Center Differences in Sedation and Analgesia Dosing During Targeted Temperature Management
Source: Neurocrit Care. 2022 Jul 28;38(1):16–25. doi: 10.1007/s12028-022-01564-6 (PMC9935704; doi:10.1007/s12028-022-01564-6)
Supplement: Supplementary file 7 — Supplementary file7 (DOCX 14 kb) [file 12028_2022_1564_MOESM7_ESM.docx]

**Supplement table 6:** Association of clinical factors, target temperature and titration of midazolam and fentanyl between 12 to 24 and 24 to 48 hours with clinical seizures, without and with center as fixed effect, in multivariate analysis.

| Patient characteristics and medication | 12 – 24 hours | 12-24 hours with center | 24-48 hours | 24-48 hours with center |
| --- | --- | --- | --- | --- |
| Age^a^ | 1.07 (0.98, 1.17) p=0.14 | 1.06 (0.97, 1.16) p=0.20 | 1.07 (0.98, 1.16) p=0.20 | 1.08 (0.98, 1.18) p=0.13 |
| Female sex | 0.88 (0.49, 1.52) p=0.60 | 0.90 (0.50, 1.60) p=0.70 | 0.93 (0.52, 1.63) p=0.80 | 0.92 (0.50, 1.66) p=0.80 |
| Witnessed arrest | 0.71 (0.37, 1.43) p=0.30 | 0.67 (0.14, 0.43) p<0.01 | 0.74 (0.38, 1.50) p=0.40 | 0.23 (0.13, 0.41) p<0.01 |
| Shockable rhythm | 0.24 (0.14, 0.40) p<0.001 | 0.24 (0.14, 0.43) p<0.001 | 0.26 (0.15, 0.43) p<0.001 | 0.23 (0.13, 0.41) p<0.001 |
| Time to ROSC^b^ | 1.02 (1.01, 1.03)  p<0.001 | 1.02 (1.01, 1.03) p<0.001 | 1.02 (1.01, 1.03)  p<0.001 | 1.02 (1.01, 1.03) p<0.001 |
| Shock on admission | 0.55 (0.24, 1.17) p=0.14 | 0.55 (0.23, 1.23) p=0.20 | 0.74 (0.33, 1.59) p=0.50 | 0.71 (0.29, 1.61) p=0.40 |
| Target temperature 36°C | 0.99 (0.65, 1.51) p>0.90 | 0.98 (0.63, 1.52) p>0.90 | 1.02 (0.67, 1.55) p>0.90 | 0.94 (0.60, 1.48) p=0.80 |
| Fentanyl equivalents | 1.40 (0.78, 2.51) p=0.20 | 1.75 (0.90, 3.42) p=0.10 | 1.22 (0.74, 2.09) p=0.40 | 1.58 (0.85, 3.03) p=0.20 |
| Midazolam equivalents | 0.54 (0.00, 17.2) p=0.80 | 0.13 (0.00, 11.2) p=0.50 | 94,3 (0.94, >100) p=0.06 | **240 (1.33, >100) p=0.04** |
| ^a^Age estimate is per 5 year intervals  ^b^ Time to ROSC estimate is per 5 minute intervals | | | | |
